# Genomic regions associated with bovine milk fatty acids in both summer and winter milk samples

**DOI:** 10.1186/1471-2156-13-93

**Published:** 2012-10-29

**Authors:** Aniek C Bouwman, Marleen HPW Visker, Johan AM van Arendonk, Henk Bovenhuis

**Affiliations:** 1 Animal Breeding and Genomics Centre, Wageningen University, P.O. Box 338, Wageningen, 6700 AH, The Netherlands

**Keywords:** Milk fatty acids, Dairy, Genome-wide association

## Abstract

**Background:**

In this study we perform a genome-wide association study (GWAS) for bovine milk fatty acids from summer milk samples. This study replicates a previous study where we performed a GWAS for bovine milk fatty acids based on winter milk samples from the same population. Fatty acids from summer and winter milk are genetically similar traits and we therefore compare the regions detected in summer milk to the regions previously detected in winter milk GWAS to discover regions that explain genetic variation in both summer and winter milk.

**Results:**

The GWAS of summer milk samples resulted in 51 regions associated with one or more milk fatty acids. Results are in agreement with most associations that were previously detected in a GWAS of fatty acids from winter milk samples, including eight ‘new’ regions that were not considered in the individual studies. The high correlation between the –log_10_(*P*-values) and effects of SNPs that were found significant in both GWAS imply that the effects of the SNPs were similar on winter and summer milk fatty acids.

**Conclusions:**

The GWAS of fatty acids based on summer milk samples was in agreement with most of the associations detected in the GWAS of fatty acids based on winter milk samples. Associations that were in agreement between both GWAS are more likely to be involved in fatty acid synthesis compared to regions detected in only one GWAS and are therefore worthwhile to pursue in fine-mapping studies.

## Background

Dairy producers are looking for ways to optimize bovine milk fat composition for human health, and to improve physical and functional properties of milk. Increasing the knowledge about the synthesis of milk fatty acids, by unravelling the genetic background of milk fatty acids, can aid in modifying bovine milk fat composition. Polymorphisms in *diacylglycerol-O-acyltransferase 1* (*DGAT1*) and *stearoyl-CoA desaturase 1* (*SCD1*) are known to have an effect on milk fatty acids, e.g. the *DGAT1* K232A polymorphism explains 40% of the genetic variation of C16:0 [1] and the *SCD1* A293V polymorphism explains 23% of the genetic variation of C16:1 [2]. However, there is still a considerable amount of genetic variation in milk fat composition that has not been assigned to polymorphisms.

Chromosomal regions associated with milk fatty acids can be detected by screening the whole genome in a genome-wide association study (GWAS). In GWAS studies many thousands of single nucleotide polymorphisms (SNPs) are being tested for associations. In general it is expected that only a small proportion of the SNPs will have a true association and only those that have an effect that is large enough will be significant. Setting a significance threshold is finding the balance between limiting the number of false positives and maintaining sufficient power. Replication of results in independent samples is a strategy to separate false positives from true associations [3,4].

In a previous GWAS we identified interesting regions of the bovine genome associated with milk fatty acids from winter milk samples [2]. To our knowledge, at present that is the only GWAS on bovine milk fatty acids. Not many populations that are large enough for GWAS have been phenotyped for milk fatty acids, because accurate measurement of milk fatty acids using gas chromatography is expensive and time consuming. In addition, genotyping a large number of individuals for a large numbers of SNPs is costly. The population that was used for our previous GWAS, based on milk samples taken in winter, has been phenotyped for milk fatty acids in a second milk sample which was taken in summer. Repeating the GWAS for milk fatty acids based on winter samples using the summer samples can confirm the previously detected associations and result in new associations.

In this study we performed a GWAS for fatty acids based on summer milk samples. This study repeats our previous GWAS for fatty acids based on winter milk samples from the same population and largely the same animals. Fatty acids from summer and winter milk are genetically similar traits and we therefore compared the regions detected in summer milk to the regions previously detected in winter milk to confirm associations.

## Methods

### Phenotypes

The phenotypes of the winter milk samples were described earlier in Bouwman et al.[2], the phenotypes of the summer milk samples will be described in detail, as well as the differences between winter and summer samples.

The fat composition of summer milk samples from 1,795 first-lactation Dutch Holstein Friesian cows was available for this study. The cows were housed on 383 commercial farms throughout the Netherlands. At least three cows were sampled per farm. The cows were between 97 and 335 days in lactation when the summer samples were taken. The pedigree of the cows was provided by CRV (Cooperative cattle improvement organization, Arnhem, the Netherlands) and consisted of 26,300 animals.

Milk fat composition was measured by gas chromatography as described in Stoop et al. [5]. Many fatty acids were measured, but only the major fatty acids are reported here: even-chain saturated fatty acids C4:0 to C18:0, even-chain (*cis9*) monounsaturated fatty acids C10:1 to C18:1, and the polyunsaturated fatty acid C18:2*cis9,trans11* (CLA). These fatty acids made up 88% of the total milk fat. The fatty acids are expressed in terms of weight-proportion of total fat weight (w/w%).

The winter and summer milk samples were taken from the same cows during the same lactation. The moment of sampling resulted in some differences between the two samples. Winter milk samples were taken from February to March 2005, when Dutch cows are mainly kept indoors and fed silage. Summer milk samples were taken from May to June 2005, when Dutch cows are often grazing for at least some part of the day. Some cows sampled in winter were not in lactation anymore during summer, therefore additional cows were sampled in summer to assure at least 3 cows per herd. The cows were on average 167 days (63-282) in lactation when the winter samples were taken, and 247 days (97-335) in lactation when the summer samples were taken.

### Statistical analysis of phenotypes

Variance components, heritabilities, and correlations between the fatty acids from summer and winter milk samples were estimated using a bivariate animal model in ASReml [6]:

(1) $$\begin{array}{l} {\text{y}}_{\text{ijklmn}}=\text{μ}+{\text{b}}_{1}{\text{*dim}}_{\text{i}}+{\text{b}}_{2}{\text{*e}}^{-0{\text{.05*dim}}_{\text{i}}}+{\text{b}}_{3}{\text{*afc}}_{\text{j}}+{\text{b}}_{4}{\text{*afc}}_{\text{j}}^{2}+\\ {\text{season}}_{\text{k}}+{\text{scode}}_{\text{l}}+{\text{herd}}_{\text{m}}+{\text{animal}}_{\text{n}}+{\text{e}}_{\text{ijklmn}}\text{,} \end{array}$$

where y was the phenotype; μ was the overall mean; dim was the covariate describing the effect of days in milk; afc was the covariate describing the effect of age at first calving; season was the fixed effect of the class of calving season (June-Aug 2004, Sept-Nov 2004, or Dec 2004-Jan 2005); scode was the fixed effect accounting for differences in genetic level between proven sire daughters and test-sire daughters, proven sires are selected and therefore their daughters might have a different genetic level than daughters of test sires; herd was the random effect of herd, distributed as N(0, I *σ*_*herd*_^2^), with identity matrix **I** and herd variance *σ*_herd_^2^; animal was the random additive genetic effect, distributed as N(0, A *σ*_*a*_^2^), with the additive genetic relationship matrix **A** and the additive genetic variance *σ*_*a*_^2^; and e was the random residual, distributed as N(0, I *σ*_*e*_^2^), with identity matrix **I** and residual variance *σ*_*e*_^2^.

### Genotypes

Blood samples were collected from the cows for DNA isolation. Of the 1,795 cows phenotyped for the summer milk sample 1,656 were successfully genotyped for 50,855 single nucleotide polymorphisms (SNPs) using a custom Infinium Array (Illumina, San Diego, CA, USA) designed by CRV. The assumed map positions of the SNPs were based on the bovine genome assembly BTAU 4.0 [7]. The average distance between SNPs was 52,452 bp. Of the 50,855 SNPs, 591 SNPs were located on the X chromosome, and 776 SNPs could not be mapped to any of the *Bos taurus* (BTA) chromosomes and were assigned to BTA 0. The SNPs on BTA 0 and the X chromosome were included in the study. Single nucleotide polymorphisms with a genotyping rate < 80% (n=392), monomorphic SNPs (n=236), and SNPs with 1-9 observations for one of the genotype classes (SNPs with such low number of observations in one of the genotype classes were excluded from further analyses to reduce the number of spurious associations) (n=5,646) were discarded from the original SNP set, resulting in the final marker set of 44,581 SNPs used for the summer GWAS.

### Ethical approval

Genomic DNA of the cows was isolated from whole blood samples of the cows. Blood samples were collected in accordance with the guidelines for the care and use of animals as approved by the ethical committee on animal experiments of Wageningen University (protocol: 200523.b).

### Genome-wide association based on summer milk samples

For the GWAS based on the summer milk samples, both phenotype and genotype data were available for 1,656 individuals. A single SNP GWAS was performed using an univariate animal in ASReml that was the same as model 1 but extended with a fixed effect for the SNP genotype.

The genome-wide FDR was based on the *P*-values from the animal model using the R package ‘qvalue’ [8]. A genome-wide FDR was calculated for each trait individually. Associations with a genome-wide FDR<0.05 were considered significant.

SNPs significant for one trait located close to each other were termed a “region”. All significant SNPs in a region might be associated with the same causal mutation. A region was defined as follows: it started at the first significant SNP on a chromosome that was followed by an additional significant SNP within 10 Mbp; the region was extended as long as another significant SNP occurred within 10 Mbp from the previous one and ended at the last significant SNP that was not followed by another significant SNP within the next 10 Mbp. Thus, each region contained at least two SNPs significant for the trait. More than one region could be present on the same chromosome when there were groups of significant SNPs located within 10 Mbp from each other but further than 10 Mbp from the other region(s) on that chromosome.

The genetic variance explained by a SNP was calculated from the estimated genotype effects from the statistical model and the observed genotype frequencies. The result was expressed as a percentage of the total additive genetic variance obtained from model 1. These percentages can be overestimated due to the so called Beavis effect, especially when the effect of a SNP is small [9]. For each trait, the proportion of genetic variance explained by the most significant SNP in a region was reported. Note that the most significant SNP in a region can differ between traits.

### Comparison of summer and winter GWAS results

The GWAS results from the winter milk samples from Bouwman et al. [2] and from the summer milk samples were compared to each other. A total of 1,564 cows were studied in both the summer and the winter GWAS, 92 cows were only studied in the summer GWAS, and 142 only in the winter GWAS.

In Bouwman et al. [2] a two-step single SNP approach was used for the GWAS of fatty acids from winter milk samples. Due to computation time, in the study by Bouwman et al. [2] only regions which showed significant associations in analyses using a general linear model were re-analysed using an animal model to account for all relations between the animals. Here we used a one-step approach using the animal model only. To make results from the previous study and the present study comparable, the GWAS based on winter milk samples was redone using an animal model for all SNPs. The Pearson correlation between the -log_10_(*P*-values) of the general linear model for winter milk samples and the animal model for winter milk samples was 0.95, which indicates that the general linear model used correctly identified the regions of interest and that the results for winter milk samples of Bouwman et al. [2] are comparable with the results presented in the current study.

For the individual GWAS studies for winter and summer milk samples a FDR<0.05 was used. A FDR<0.05 is stringent, especially when looking for agreement of results. Therefore, a SNP was qualified as associated with both summer and winter milk fatty acids when the FDR threshold was smaller than 0.20 in both GWAS studies. We choose a FDR threshold of 20%, because the FDR of agreement between the two GWAS studies would then be 4% (20%*20%) if the studies were independent [10]. SNPs in agreement were reported when at least more than one SNP was in agreement between the summer and winter GWAS in a region (were region was defined as above) or when the SNP was in agreement between the summer and winter GWAS for more than one trait.

## Results

Table 1 shows that the phenotypic variation of milk fatty acids was larger in summer than in winter. Summer milk samples contained more long chain fatty acids (C18:0, C18:1, and CLA) and less C16:0 than winter milk samples (Table 1). Phenotypic correlations between fatty acids of summer and winter milk samples ranged between 0.36 and 0.67 (Table 2). Genetic correlations between fatty acids of summer and winter milk samples ranged between 0.77 and 1.00 (Table 2). Genetic correlations between summer and winter samples of C4:0, C6:0, C12:0, C18:0, C10:1, C12:1, C14:1, C16:1, and C18:1 (ranging between 0.90-1) were not significantly different from one (Table 2). The genetic correlations for C8:0, C10:0, C14:0, C16:0, and CLA were significantly different from one but showed strong positive correlations (0.77-0.94, Table 2). Herd correlations between fatty acids of summer and winter milk samples ranged between 0.16 and 0.54 (Table 2).

**Table 1:** **Mean (in w/w%), phenotypic variance** (*σ*_*p*_^2^ = *σ*_*a*_^2^ + *σ*_*herd*_^2^ + *σ*_*e*_^2^)**, intra-herd heritability**$\left(h_{\mathrm{IH}}^{2}=\frac{\sigma_{a}^{2}}{\sigma_{a}^{2}+\sigma_{e}^{2}}\right)$**, and proportion of variance due to herd**$\left(Herd=\frac{\sigma_{\mathrm{herd}}^{2}}{\sigma_{a}^{2}+\sigma_{\mathrm{herd}}^{2}+\sigma_{e}^{2}}\right)$**for the fatty acids of summer and winter milk samples, with their standard errors in subscript**

**Trait**	**Summer n=1,795**				**Winter n=1,905**			
	**Mean**	***σ***_***P***_^**2**^	***h***_***IH***_^**2**^	**Herd**	**Mean**	***σ***_***P***_^**2**^	***h***_***IH***_^**2**^	**Herd**
C4:0	3.52_0.35_	0.1267_0.01_	0.37_0.09_	0.24_0.02_	3.50_0.27_	0.0775_0.00_	0.43_0.09_	0.16_0.02_
C6:0	2.17_0.21_	0.0435_0.00_	0.41_0.09_	0.18_0.02_	2.22_0.17_	0.0278_0.00_	0.48_0.10_	0.16_0.02_
C8:0	1.32_0.17_	0.0288_0.00_	0.41_0.09_	0.19_0.02_	1.37_0.14_	0.0202_0.00_	0.62_0.11_	0.20_0.02_
C10:0	2.87_0.46_	0.2240_0.01_	0.56_0.10_	0.19_0.02_	3.03_0.43_	0.2009_0.01_	0.74_0.11_	0.23_0.02_
C12:0	3.78_0.73_	0.5550_0.03_	0.52_0.10_	0.40_0.03_	4.11_0.69_	0.5041_0.02_	0.64_0.11_	0.43_0.03_
C14:0	11.15_1.06_	1.1560_0.05_	0.51_0.10_	0.34_0.03_	11.61_0.92_	0.8953_0.04_	0.58_0.10_	0.17_0.02_
C16:0	29.17_3.50_	12.4400_0.60_	0.36_0.10_	0.50_0.03_	32.59_2.83_	8.2030_0.35_	0.37_0.10_	0.30_0.03_
C18:0	9.88_1.77_	3.1540_0.13_	0.18_0.07_	0.30_0.03_	8.72_1.42_	1.9770_0.07_	0.24_0.07_	0.19_0.02_
C10:1	0.35_0.07_	0.0051_0.00_	0.48_0.10_	0.25_0.03_	0.37_0.07_	0.0044_0.00_	0.33_0.08_	0.10_0.02_
C12:1	0.11_0.03_	0.0010_0.00_	0.47_0.10_	0.30_0.03_	0.12_0.03_	0.0008_0.00_	0.37_0.08_	0.21_0.02_
C14:1	1.38_0.28_	0.0754_0.00_	0.46_0.09_	0.15_0.02_	1.36_0.26_	0.0614_0.00_	0.33_0.08_	0.07_0.02_
C16:1	1.40_0.30_	0.0938_0.00_	0.39_0.09_	0.09_0.02_	1.44_0.32_	0.1047_0.00_	0.42_0.09_	0.07_0.02_
C18:1	20.56_2.80_	7.8000_0.34_	0.37_0.10_	0.34_0.03_	18.18_2.04_	4.1790_0.17_	0.28_0.09_	0.29_0.03_
CLA	0.56_0.28_	0.0796_0.00_	0.28_0.09_	0.58_0.02_	0.39_0.11_	0.0130_0.00_	0.44_0.10_	0.51_0.02_

**Table 2:** **Phenotypic (r**_**p**_**), additive genetic (r**_**a**_**), herd (r**_**herd**_**), and residual correlation (r**_**e**_**) between winter and summer milk samples, with their standard errors (se)**

**Trait**	**r**_**p**_^**1**^	**se**	**r**_**a**_^**2**^	**se**	**r**_**herd**_	**se**	**r**_**e**_	**se**
C4:0	0.48	0.02	0.94^ns^	0.06	0.31	0.08	0.25	0.09
C6:0	0.55	0.02	0.95^ns^	0.05	0.42	0.08	0.29	0.09
C8:0	0.52	0.02	0.93 ^*^	0.05	0.39	0.08	0.17	0.14
C10:0	0.56	0.02	0.94 ^*^	0.04	0.41	0.07	-0.03	0.26
C12:0	0.54	0.02	0.98^ns^	0.03	0.54	0.05	-0.06	0.21
C14:0	0.52	0.02	0.94 ^*^	0.04	0.37	0.07	0.14	0.15
C16:0	0.42	0.03	0.77^**^	0.11	0.20	0.06	0.47	0.07
C18:0	0.45	0.02	0.90^ns^	0.10	0.26	0.08	0.41	0.05
C10:1	0.44	0.02	1.00^ns^	0.03	0.31	0.10	0.15	0.10
C12:1	0.49	0.02	1.00^ns^	0.03	0.37	0.07	0.21	0.10
C14:1	0.61	0.02	1.00^ns^	0.02	0.16	0.14	0.46	0.06
C16:1	0.67	0.02	0.97^ns^	0.03	0.19	0.17	0.53	0.06
C18:1	0.41	0.03	0.92^ns^	0.08	0.19	0.07	0.33	0.07
CLA	0.36	0.03	0.81^**^	0.11	0.30	0.06	0.24	0.08

^1^ r_p_ is based on *σ*_*p*_^2^ = *σ*_*a*_^2^ + *σ*_*herd*_^2^ + *σ*_*e*_^2^.

^2^ Superscripts indicate whether the genetic correlation differs significantly from 0.995 (~1), where **P-value < 0.01, * P-value ≤ 0.05 and ns = non-significant, i.e., P > 0.05.

### Genome-wide association based on summer milk samples

**Figure 1:**
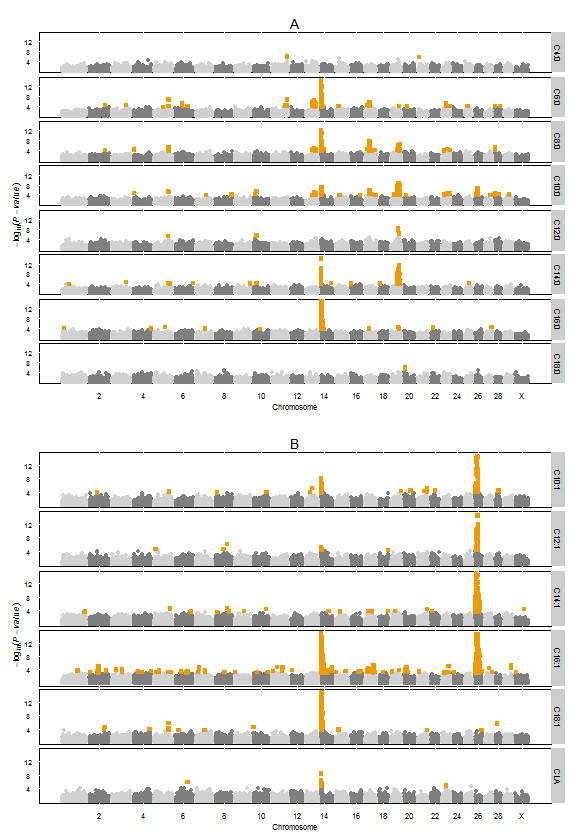
**Genome-wide association plots for bovine milk fatty acids of summer milk samples.** Genome-wide plots of -log_10_ (*P*-values) (y-axis) for association of SNPs with saturated fatty acids (**A**) and unsaturated fatty acids (**B**). The genomic position is represented along the x-axis and chromosome numbers are given on the x-axis. The dashed horizontal lines represent the 0.05 false discovery rate thresholds. The orange squares represent the significant SNPs (FDR<0.05). The y-axis are cut off at -log_10_(*P*-value) of 15.

Figure 1 shows the genome-wide plots of -log_10_(*P*-values) of the GWAS of the fatty acids based on the summer milk samples. In total, 51 regions were associated with one or more fatty acids. Table 3 gives all detected regions and the percentage of the total additive genetic variation explained by the most significant SNP in that region for each of the fatty acids (for more detailed information about those most significant SNPs see Additional file 1). The most significant SNPs per region explained 2.2% up to 50.1% of the total additive genetic variation (Table 3). When all these most significant SNPs per region per fatty acid were analysed simultaneously they explained between 5.5% for C4:0 and 92.5% for C16:1 (Table 3). Three regions with major effects were associated with multiple fatty acids: BTA 14, BTA 19, and BTA 26. First we will describe the results for these three regions with major effects and then for the other regions associated with more than one fatty acid. Regions associated with only one fatty acid are given in Table 3 but will not be described here.

**Table 3:** Regions significantly associated with fatty acids of the summer milk samples and the percentage of the total additive genetic variance explained by the most significant SNP in that region

**Region**^**1**^	**Start**	**End**	**Trait**														**#Traits/**
	**(Mbp)**	**(Mbp)**	**C4:0**	**C6:0**	**C8:0**	**C10:0**	**C12:0**	**C14:0**	**C16:0**	**C18:0**	**C10:1**	**C12:1**	**C14:1**	**C16:1**	**C18:1**	**CLA**	**region**
1a	105.1	106.3												3.3			1
1b	153.5	154.8											2.5				1
2a	56.3	69.5												4.0			1
2b	106.2	113.9													5.0		1
2c	118.9	118.9												3.0			1
3	72.6	72.6												3.5			1
4a	60.0	60.0												2.8			1
4b	121.8	123.7												3.2			1
5a	9.1	12.5										4.0					1
5b	36.0	36.1												3.4			1
5c	96.3	108.8		5.3	4.9	3.8		2.8			3.2		3.2	2.7	6.5		8
6a	40.6	40.8												2.9			1
6b	44.8	44.8		4.0													1
6c	76.9	85.0												2.8			1
6d	85.3	85.3		3.0													1
6e	105.1	106.1											2.9				1
7a	14.9	22.2												3.7			1
7b	64.0	64.2												3.0			1
10a	9.8	11.0				2.9											1
10b	22.0	22.0				3.0	3.9										2
10c	86.1	89.9									3.1						1
11a	69.9	74.3												3.6			1
11b	95.1	107.3	5.5	5.2													2
13	41.4	68.4		5.5	4.6	3.2											3
14a	0.0	18.9		14.1	11.0	4.2		8.1	47.4		7.1	4.6		31.1	50.1	10.5	10
14b	44.2	50.9												3.6			1
14c	69.4	77.0												4.1			1
15a	20.9	20.9													3.6		1
15b	64.1	72.9												3.0			1
16a	2.1	3.7						2.2									1
16b	51.2	68.0												3.0			1
17a	15.0	23.9		3.3	3.9									3.7			3
17b	24.7	24.7											3.0				1
17c	28.6	34.3		5.3	8.2	4.1											3
17d	49.6	68.7											3.3	4.4			2
17e	74.0	74.0				2.2											1
19a	6.1	6.1												2.9			1
19b	37.3	62.3			6.0	5.7	6.3	12.3	4.3								5
20	8.5	26.1		2.8						15.6				4.0			3
21	63.8	65.2									4.2						1
22a	11.8	11.8											2.9				1
22b	16.2	16.2							3.7								1
23a	15.5	23.8												2.7			1
23b	26.3	33.2		4.3													1
23c	42.7	48.5												4.1			1
26	1.4	39.0				4.5					20.6	15.0	46.4	30.7			5
27	42.7	47.6												2.6			1
28	3.1	3.1			4.9	3.1											2
29a	32.7	32.7				2.6											1
29b	44.3	44.3												3.5			1
X	63.1	63.1											3.9				1
Sum			5.5	52.8	43.6	39.3	10.2	25.4	55.4	15.6	38.3	23.6	68.0	145.3	65.2	10.5	
All SNPs in model^2^			5.5	28.4	35.6	29.3	6.5	21.4	51.2	15.6	37.1	22.0	61.8	92.5	63.6	10.5	
# Regions/trait			1	10	7	11	2	4	3	1	5	3	8	27	4	1	87

^1^ A region starts at the first significant SNP and proceeds if the next significant SNP is positioned within the next 10 Mbp on the same chromosome, ending at the position of the last significant SNP matching this requirement, with a minimum of 2 significant SNPs per trait in a region. The number of the region stands for the BTA number plus an a, b, c, d or e indicating different regions within a BTA.

^2^ Percentage of the total additive genetic variance explained by all the most significant SNPs per region together. This was analyzed with the animal model and all the most significant SNPs per region simultaneous in the model.

The association detected on BTA 14, between 0 and 18.9 Mbp (region 14a), with C6:0, C8:0, C14:0, C16:0, C16:1, C18:1, and CLA was most significant for three SNPs located in the *DGAT1* gene at 0.4 Mbp (including the two SNPs underlying the *DGAT1 K232A* dinucleotide polymorphism). The *DGAT1* SNPs explained 8.1-50.1% of the total additive genetic variation of these fatty acids (Table 3). The association detected on BTA 14 with C10:0 and C10:1 was also significant for the SNPs in the *DGAT1* gene. However, for these fatty acids the SNPs in the *DGAT1* gene were not the most significant ones in this region on BTA 14. The most significant SNPs were located at 3.6 Mbp for C10:0 and 3.0 Mbp for C10:1. The association detected on BTA 14 with C12:1 was not significant for the SNPs in the *DGAT1* gene (-log_10_(*P*-value)=1.89). The association detected on BTA 14 with C12:1 was most significant for a SNP located at 3.2 Mbp. After correcting C10:0, C10:1, and C12:1 for the effect of the *DGAT1 K232A* polymorphism these most significant SNPs remained significant. The linkage disequilibrium (LD) between these SNPs and the *DGAT1 K232A* SNPs was moderate (r^2^ = 0.14-0.34).

The association detected on BTA 19, between 37.3-62.3 Mbp (region 19b), with C8:0, C10:0, C12:0, C14:0, and C16:0 was most significant around 46 Mbp for C10:0, C12:0, and C16:0; around 52 Mbp for C14:0; and around 58 Mbp for C8:0. The most significant SNPs explained 4.3-12.3% of the total additive genetic variation of these fatty acids (Table 3).

The association detected on BTA 26, between 1.4 and 39.0 Mbp, with C10:0, C10:1, C12:1, C14:1, and C16:1 was most significant near the *SCD1* gene. The *SCD1* gene is not mapped on the BTAU 4.0 [7], but is mapped at 21 Mbp on BTA 26 according to the UMD 3.0 map [11]. Two SNPs located in the *SCD1* gene (including the *SCD1 A293V* polymorphism) were the most significant SNPs for the traits associated with the region on BTA 26. The *SCD1* SNPs explained 4.5% of the genetic variation of C10:0 and 15-46.4% of the total additive genetic variation of the medium chain unsaturated fatty acids (Table 3).

Beside these three regions with major effects, nine additional regions were associated with more than one fatty acid (Table 3). Region 5c was associated with C6:0, C8:0, C10:0, C14:0, C10:1, C14:1, C16:1, and C18:1. Region 10b was associated with C10:0, and C12:0. Region 11b was associated with C4:0, and C6:0. The region on BTA 13 was associated with C6:0, C8:0, and C10:0. On BTA 17 there were three regions associated with multiple traits: region 17a between 15.0 and 23.9 Mbp was associated with C6:0, C8:0, and C16:1; region 17c between 28.6 and 34.3 Mbp was associated with C6:0, C8:0, and C10:0; region 17d between 49.6 and 68.7 Mbp was associated with C14:1, and C16:1. The region on BTA 20 was associated with C6:0, C18:0, and C16:1. The region on BTA 28 was associated with C8:0, and C10:0.

Some SNPs located on BTA 0 were also significant. Blasting these SNPs against the UMD 3.0 map showed that they were mainly located in regions that already showed significant effects, such as region 14a, 19b and 26.

### Comparison of summer and winter GWAS results

**Figure 2:**
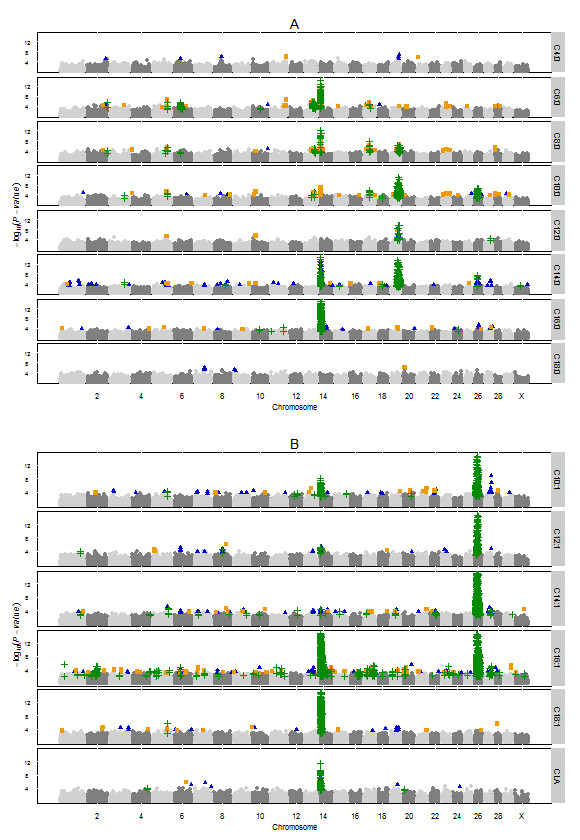
**Results from winter and summer genome-wide association studies for bovine milk fatty acids combined in Manhattan-plots.** Genome-wide plots of -log_10_ (*P*-values) (y-axis) for association of SNPs with saturated fatty acids (**A**) and unsaturated fatty acids (**B**). The genomic position is represented along the x-axis and chromosome numbers are given on the x-axis. The orange squares represent SNPs only significant for the summer milk samples (FDR_summer_<0.05, FDR_winter_≥0.20). Blue triangles represent SNPs only significant for the winter milk samples (FDR_winter_<0.05, FDR_summer_≥0.20). Green addition signs represent SNPs that were detected significant in both milk samples (FDR<0.20 in both studies). Note that each SNP is represented twice in this figure, once at the -log_10_(*P*-value) for the summer GWAS and once at the -log_10_(*P*-value) for the winter GWAS. The y-axis are cut off at -log_10_(*P*-value) of 15.

Figure 2 shows two -log_10_(*P*-values) for each SNP, one for the summer (significant SNPs are orange squares in Figure 2) and one for the winter (significant SNPs are blue triangles in Figure 2) GWAS. The -log_10_(*P*-values) of SNPs that had a FDR<0.20 in both the winter and summer GWAS are indicated with green addition signs and show the regions that were found for both samples. Table 4 gives an overview of the regions associated with the summer milk fatty acids that were in agreement with the previous study of winter milk fatty acids. Only the regions that showed agreement between the summer and winter GWAS for more than one SNP or more than one trait are reported in Table 4, resulting in 34 regions.

**Table 4:** Regions associated (FDR<0.20) with milk fatty acids in both the summer and winter GWAS

**Region**^**1**^	**Start (Mbp)**	**End (Mbp)**	**Trait**													
			**C4:0**	**C6:0**	**C8:0**	**C10:0**	**C12:0**	**C14:0**	**C16:0**	**C18:0**	**C10:1**	**C12:1**	**C14:1**	**C16:1**	**C18:1**	**CLA**
1	134.0	134.0										1	1			
2a	31.9	32.5												3		
2b	56.3	69.5												19		
2c	139.5	139.5		1	1											
3	97.9	97.9				1		1								
4	121.8	123.7											1	2		
5a	24.4	25.3												2		
5b	33.9	36.1												3		
5c	100.0	101.1		2	2	2					2				1	
5d	108.8	108.8											2			
6	40.0	59.2		11	1									6		
7	64.1	64.1												4		
9	66.2	66.2												2		
10	7.4	7.4												3		
12	52.4	52.5									3		3			
13	46.1	68.4		28	10	3					1					
14a	0.0	18.9		36	15			45	256		42	12	2	375	233	50
14b	32.7	32.7									1		1			
14c	40.8	50.9												4		
14d	73.0	76.5												2		
16	46.7	68.0												15		
17a	13.5	21.0											1	6		
17b	31.4	34.7		1	4	5								1		
17c	43.4	43.4		1	1											
17d	56.4	69.8												14		
18	22.8	22.8				2		2								
19a	6.1	7.6												4		
19b	32.9	64.9			20	64	11	103					2	2		
20	8.6	12.3												3		1
22	36.1	42.4											5			
23	42.7	48.5												3		
24	39.2	39.2							2							
26	2.5	40.8				29		6		1	133	61	260	202		
27	30.2	47.6					1						4	7		
Total # SNPs in agreement			0	80	54	106	12	157	258	1	182	74	282	682	234	51

^1^ Region number corresponds to the BTA number plus an a, b, c, or d indicating different regions within a BTA.

The number of SNPs in agreement between summer and winter GWAS in the region are given and only regions txhat had more than one SNP or more than one trait in agreement between summer and winter GWAS are reported here.

Three regions with major effects, BTA 14, 19, and 26, were found for both summer and winter milk fatty acids. These regions were highly significant in the GWAS based on winter milk samples and were therefore expected to be found for the summer milk samples too. More interesting are the additional regions that were found in both GWAS studies and especially the eight regions (1, 2a, 3, 5a, 10, 14b, 17c, and 24 (Table 4)) that were not reported for the individual studies based on winter or on summer milk samples because their FDR was between 0.05 and 0.20. In some regions agreement between the summer and winter GWAS was based on a single SNP but in other regions agreement was based on multiple SNPs. Also, some regions were associated with multiple fatty acids. We will report here the regions found to be associated with more than two fatty acids in both GWAS studies: regions 5c, 6, 13, 17b, and 27 (Table 4).

On BTA 5 the associations in region 5c with C6:0, C8:0, C10:0, C10:1, and C18:1 were in agreement with the winter GWAS [2]. There are no obvious candidate genes located in this region. In both GWAS studies this region was also associated with C14:0, but different SNPs were significant in the two studies (see Figure 2). The agreement between summer and winter GWAS for C14:1 seems to be in a separate region, region 5d at 108.8 Mbp.

On BTA 6 the associations with C6:0, C8:0, and C16:1 (Table 4) were in agreement with the winter GWAS [2]. This region contains the candidate gene *peroxisome proliferator-activated receptor gamma, coactivator 1 alpha* (*PPARGC1A*). Our SNP set contained 10 SNPs located in *PPARGC1A*, however, none of these SNPs were significant in winter nor in summer, but SNPs around (73,865bp before and 797,923bp after) the gene showed association in both the summer and winter GWAS. In the winter GWAS the region was also associated with C12:1 and C14:1, but this was not the case in the summer GWAS.

On BTA 13 the associations with C6:0, C8:0, C10:0, and C10:1 (Table 4) were in agreement with the winter GWAS [2]. The region on BTA 13 was associated with short chain fatty acids. A possible candidate gene in this region is *acyl-CoA synthetase short-chain family member 2*, which activates acetate for *de novo* fatty acid synthesis [12]. In the winter GWAS the region was also associated with C14:1 and C16:1, but this was not the case in the summer GWAS.

On BTA 17 the associations in region 17b with C6:0, C8:0, C10:0, and C16:1 (Table 4) were in agreement with the winter GWAS [2]. There are no obvious candidate genes located in this region.

On BTA 27 the associations with C12:0, C14:1, and C16:1 (Table 4) were in agreement with the winter GWAS [2]. This region contains the candidate gene *1-acylglycerol-3-phosphate O-acyltransferase 6*, which is involved in attaching fatty acids on the second position of the triglyceride backbone. In the winter GWAS this region was also associated with C14:0, C16:0, C10:1, and C12:1, but this was not the case in the summer GWAS.

**Figure 3:**
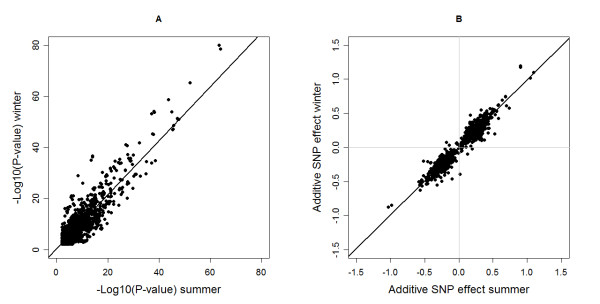
**Significance level (A) and additive SNP effects expressed in phenotypic standard deviation**$\left(\sigma_{p}=\sqrt{\sigma_{a}^{2}+\sigma_{e}^{2}}\right)$**(B) of the SNPs that were found significant in both the summer and winter GWAS (FDR<0.20).**

Besides agreement of association for both summer and winter milk fatty acids it is also interesting to see how well the significance levels and effects of SNPs from the summer and winter GWAS correlate. High correlation implies that the effect of the QTL is similar in winter as in summer and, thus, that there is no genotype by season interaction for the regions that were found in both GWAS studies. Winter and summer -log_10_(*P*-values) of SNPs that had a FDR<0.20 in both GWAS studies are plotted in Figure 3A and showed a correlation of 0.89. Winter and summer additive SNP effects of SNPs that had a FDR<0.20 in both GWAS studies, expressed in phenotypic standard deviation, are plotted in Figure 3B and show a correlation of 0.97.

## Discussion

The aim of this study was to perform a GWAS of bovine fatty acids based on summer milk samples and to compare them to previous results of a GWAS of fatty acids based on winter milk samples [2]. For this GWAS we used different milk samples from largely the same set of cows with the same genotypes at a different stage of the same lactation. The main difference between the two seasons was the herd management, including feeding. In winter, all herds were kept indoors and fed silage, while in summer about half of the herds were grazing outside for at least part of the day and a few other herds were fed fresh grass. Diets of cows including fresh grass are known to alter milk fat composition e.g. [13,14]. This was reflected in our results: summer milk contained more long chain fatty acids and less C16:0 compared to winter milk. The phenotypic correlations for the different fatty acids between summer and winter samples ranged between 0.36-0.67 (Table 2), indicating that the summer samples provide additional information compared to the winter samples. Genetic correlations between summer and winter samples of C4:0, C6:0, C12:0, C18:0, C10:1, C12:1, C14:1, C16:1, and C18:1 (ranging between 0.90-1, Table 2) showed that these fatty acids are genetically the same trait in summer and winter [15]. The genetic correlations for C8:0, C10:0, C14:0, C16:0, and CLA were significantly different from one [15] but showed strong positive correlations (0.77-0.94, Table 2), suggesting that also for these fatty acids summer and winter samples have most genetic variation in common. It is important here to consider that strong positive genetic correlations are required to ensure that the traits have the same genetic background. However, for the summer milk sample to provide additional information to our previous GWAS phenotypic correlations should be weak.

This GWAS of fatty acids based on summer milk samples shows agreement with most associations detected in our previous GWAS of fatty acids based on winter milk samples [2]. Three regions with major effects detected in the winter GWAS [2] were also found in the summer GWAS. On BTA 14 a dinucleotide polymorphism in *DGAT1* is causing the major effect. The *DGAT1 K232A* polymorphism is known to be associated with fat content and composition, so our results are in line with other studies [1,16,17]. The rather large region associated with the short and medium chain saturated milk fatty acids on BTA 19 confirm previous linkage studies [18-20]. Several candidate genes related to fat synthesis are located in this region, e.g. *ATP citrate lyase*, *sterol regulatory element-binding transcription factor 1*, *signal transducer and activator of transcription 5A*, *growth hormone*, and *fatty acid synthase*. There might be more than one QTL in this region given the size of this region and the many candidate genes, but the actual polymorphism(s) causing the effect(s) has not yet been identified. On BTA 26 a polymorphism in *SCD1* is causing the major effect. The gene *SCD1* is known to be associated with medium-chain unsaturated fatty acids, so our results are in line with other studies [16,21-24].

Our results from both GWAS studies also suggest that there are additional QTL on BTA 14 besides *DGAT1* that were associated with fatty acids. These additional QTL on BTA 14 were located at 3.0-3.8 Mbp, 32.7 Mbp, 40.8-50.9 Mbp, and 73.0-76.5 Mbp, and confirm detected QTL for milk production traits in linkage analyses reviewed in [25]. Candidate genes for these regions might be *corticotropin releasing hormone* at 30.5 Mbp, *fatty acid binding protein 5* at 41.9 Mbp, and *fatty acid binding protein 4* at 42.0 Mbp.

The three highly significant regions with major effects mentioned above were expected to be found in both GWAS studies. More interesting are the additional regions that were found in both GWAS studies such as the regions associated with more than two fatty acids: region 5c, 6, 13, 17b, and 27. Also worthwhile to mention are the ‘new’ regions that had a suggestive FDR between 5-20% and were not considered in the individual studies based on winter or on summer milk samples only, but became of interest because they were found in both studies. There are eight ‘new’ regions like this: region 1, 2a, 3, 5a, 10, 14b, 17c, and 24 (Table 4).

There are two different confirmation strategies regarding GWAS: replication and validation. Replication studies are meant to confirm that the actual association is a true association and should therefore be based on samples from the same population with minimal systematic differences [26,27]. Validation studies are meant to see if the association can be generalized over different populations and should therefore be based on samples from a different population, where this population can be different concerning genetic background, phenotype definition, sampling strategy, and time point of investigation [26,27]. A correctly performed replication is more likely to be successful in finding the same association again than validation, however, when an association is validated the associated SNP is probably closer to the actual polymorphism. In literature, replication and validation are often used interchangeably which complicates the interpretation of the results, especially when a study is called replication study but population, phenotype or study design are too different from the original study to be a replication study. Our GWAS has elements of a replication as well as of a validation study; it met criteria for a replication such as sufficient sample size, phenotypes were measured using the same method, same set of markers and a very similar population was used. It also met criteria for validation because the phenotypes were measured at different time points. The difference in season of measuring the phenotype provides additional information. Ideally an independent population of cows should have been sampled, but this was practically not feasible.

Replication of the study with largely the same set of animals and the same genotypes led to minor differences in LD and allele frequencies between studies, therefore it is more likely to confirm previously detected results [10]. However, spurious associations due to population structure or genotyping errors are more likely to be detected twice using the same set of animals and genotypes.

Agreement between the two GWAS studies was based on a FDR threshold of 20% in each study. If the winter and summer GWAS would be independent, the FDR of a region found in both studies would be 4% (20%*20%) [10]. A FDR of 4% gives enough reason to investigate such a region further. Lowering the threshold from 5% to 20% FDR resulted in the eight ‘new’ regions mentioned above, besides the regions that were already discovered in one of the individual studies and were in agreement with the other.

Even though we used largely the same set of animals, same genotypes, same phenotype measurement and a lower threshold for agreement between summer and winter GWAS not all regions were found in both summer and winter GWAS. This can be due to genotype by season interaction, due to lack of power (false negative QTL) or because these QTL were false positive QTL. It is not possible to determine which of these three reasons apply. However, the genetic correlations indicated that fatty acids are genetically similar traits in summer and winter, which suggests that genotype by season interaction may have only a small effect on the results. So lack of agreement between summer and winter GWAS is either due to lack of power or false positives.

## Conclusions

This GWAS of fatty acids based on summer milk samples is in agreement with most associations that were previously detected in a GWAS of fatty acids based on winter milk samples. Lowering the FDR threshold from 5% in individual studies to 20% for agreement between both the summer and winter GWAS led to eight ‘new’ regions that were not considered in the individual studies, but had a suggestive FDR between 5-20% in both studies. It is more likely that genomic regions are involved in fatty acid synthesis when associations are found in both summer and winter GWAS compared to regions detected in only one GWAS. Detected associations that were in agreement between summer and winter GWAS are therefore worthwhile to pursue in fine-mapping studies.

## Authors’ contributions

ACB carried out the analysis, prepared and drafted the manuscript. MHPWV helped to draft and edit the manuscript. JAMA initiated and established the overall project design. HB participated in the design of the study, the coordination and helped to draft the manuscript. All authors read and approved the final manuscript.

## Supplementary Material

Additional 1 **Table S1.** Most significant SNP per trait for each region significantly associated with fatty acids of the summer milk sample (corresponding to table 3), SNP position, significance level and the percentage of total additive genetic variance explained by the SNP. Click here for file

## References

[B1] Schennink A Stoop WM Visker MHPW Heck JML Bovenhuis H Van Der Poel JJ Van Valenberg HJF Van Arendonk JAM DGAT1 underlies large genetic variation in milk-fat composition of dairy cows Anim Genet 2007 38 5 467 473 10.1111/j.1365-2052.2007.01635.x 17894561

[B2] Bouwman A Bovenhuis H Visker M Van Arendonk J Genome-wide association of milk fatty acids in Dutch dairy cattle BMC Genet 2011 12 1 43 21569316 10.1186/1471-2156-12-43 PMC3120725

[B3] Chanock SJ Manolio T Boehnke M Boerwinkle E Hunter DJ Thomas G Hirschhorn JN Goncalo A Altshuler D Bailey-Wilson JE Replicating genotype–phenotype associations Nature 2007 447 7145 655 660 10.1038/447655a 17554299

[B4] van den Oord EJCG Controlling false discoveries in genetic studies Am J Med Genet 2008 147B 5 637 644 10.1002/ajmg.b.30650 18092307

[B5] Stoop WM van Arendonk JAM Heck JML van Valenberg HJF Bovenhuis H Genetic Parameters for Major Milk Fatty Acids and Milk Production Traits of Dutch Holstein-Friesians J Dairy Sci 2008 91 1 385 394 10.3168/jds.2007-0181 18096963

[B6] Gilmour AR Gogel BJ Cullis BR Thompson R ASReml User Guide Release 2.0 Hemel Hempstead, HP1 1ES 2006 VSN International Ltd, UK

[B7] Liu Y Qin X Song X-Z Jiang H Shen Y Durbin KJ Lien S Kent M Sodeland M Ren Y Bos taurus genome assembly BMC Genomics 2009 10 1 180 10.1186/1471-2164-10-180 19393050 PMC2686734

[B8] Storey JD Tibshirani R Statistical significance for genomewide studies Proc Natl Acad Sci 2003 100 16 9440 9445 10.1073/pnas.1530509100 12883005 PMC170937

[B9] Beavis WD AH P QTL analyses: power precision and accuracy Molecular dissection of complex traits 1998 CRC Press, New York 145 162

[B10] Liu Y-J Papasian CJ Liu J-F Hamilton J Deng H-W Is Replication the Gold Standard for Validating Genome-Wide Association Findings? PLoS One 2008 3 12 e4037 10.1371/journal.pone.0004037 19112512 PMC2605260

[B11] Zimin A Delcher A Florea L Kelley D Schatz M Puiu D Hanrahan F Pertea G Van Tassell C Sonstegard T A whole-genome assembly of the domestic cow, Bos taurus Genome Biology 2009 10 4 R42 10.1186/gb-2009-10-4-r42 19393038 PMC2688933

[B12] Bionaz M Loor J Gene networks driving bovine milk fat synthesis during the lactation cycle BMC Genomics 2008 9 1 366 10.1186/1471-2164-9-366 18671863 PMC2547860

[B13] Fievez V Vlaeminck B Dhanoa MS Dewhurst RJ Use of Principal Component Analysis to Investigate the Origin of Heptadecenoic and Conjugated Linoleic Acids in Milk J Dairy Sci 2003 86 12 4047 4053 10.3168/jds.S0022-0302(03)74016-8 14740843

[B14] Smith S Witkowski A Joshi AK Structural and functional organization of the animal fatty acid synthase Prog Lipid Res 2003 42 4 289 317 10.1016/S0163-7827(02)00067-X 12689621

[B15] Duchemin S Bovenhuis H Stoop WM Bouwman AC Van Arendonk JAM Visker MHPW Genetic correlation between composition of bovine milk fat in winter and summer, and DGAT1 and SCD1 by season interactions J Dairy Sci 2012 in press 10.3168/jds.2012-5454 23127906

[B16] Conte G Mele M Chessa S Castiglioni B Serra A Pagnacco G Secchiari P Diacylglycerol acyltransferase 1, stearoyl-CoA desaturase 1, and sterol regulatory element binding protein 1 gene polymorphisms and milk fatty acid composition in Italian Brown cattle J Dairy Sci 2010 93 2 753 763 10.3168/jds.2009-2581 20105547

[B17] Grisart B Coppieters W Farnir F Karim L Ford C Berzi P Cambisano N Mni M Reid S Simon P Positional Candidate Cloning of a QTL in Dairy Cattle: Identification of a Missense Mutation in the Bovine DGAT1 Gene with Major Effect on Milk Yield and Composition Genome Res 2002 12 2 222 231 10.1101/gr.224202 11827942

[B18] Morris C Cullen N Glass B Hyndman D Manley T Hickey S McEwan J Pitchford W Bottema C Lee M Fatty acid synthase effects on bovine adipose fat and milk fat Mamm Genome 2007 18 1 64 74 10.1007/s00335-006-0102-y 17242864

[B19] Schennink A Bovenhuis H Léon-Kloosterziel KM Van Arendonk JAM Visker MHPW Effect of polymorphisms in the FASN, OLR1, PPARGC1A, PRL and STAT5A genes on bovine milk-fat composition Anim Genet 2009 40 6 909 916 10.1111/j.1365-2052.2009.01940.x 19719788

[B20] Stoop WM Schennink A Visker MHPW Mullaart E van Arendonk JAM Bovenhuis H Genome-wide scan for bovine milk-fat composition. I. Quantitative trait loci for short- and medium-chain fatty acids J Dairy Sci 2009 92 9 4664 4675 10.3168/jds.2008-1966 19700730

[B21] Kgwatalala PM Ibeagha-Awemu EM Hayes JF Zhao X Stearoyl-CoA desaturase 1 3′UTR SNPs and their influence on milk fatty acid composition of Canadian Holstein cows J Anim Breed Genet 2009 126 5 394 403 10.1111/j.1439-0388.2008.00796.x 19765166

[B22] Mele M Conte G Castiglioni B Chessa S Macciotta NPP Serra A Buccioni A Pagnacco G Secchiari P Stearoyl-Coenzyme A Desaturase Gene Polymorphism and Milk Fatty Acid Composition in Italian Holsteins J Dairy Sci 2007 90 9 4458 4465 10.3168/jds.2006-617 17699067

[B23] Moioli B Contarini G Avalli A Catillo G Orrù L De Matteis G Masoero G Napolitano F Short Communication: Effect of Stearoyl-Coenzyme A Desaturase Polymorphism on Fatty Acid Composition of Milk J Dairy Sci 2007 90 7 3553 3558 10.3168/jds.2006-855 17582140

[B24] Schennink A Heck JML Bovenhuis H Visker MHPW van Valenberg HJF van Arendonk JAM Milk Fatty Acid Unsaturation: Genetic Parameters and Effects of Stearoyl-CoA Desaturase (SCD1) and Acyl CoA: Diacylglycerol Acyltransferase 1 (DGAT1) J Dairy Sci 2008 91 5 2135 2143 10.3168/jds.2007-0825 18420645

[B25] Wibowo T Gaskins C Newberry R Thorgaard G Michal J Jiang Z Genome Assembly Anchored QTL Map of Bovine Chromosome 14 Int J Biol Sci 2008 4 6 406 414 19043607 10.7150/ijbs.4.406 PMC2586679

[B26] Igl BW König IR Ziegler A What Do We Mean by ‘Replication’ and ‘Validation’ in Genome-Wide Association Studies? Hum Hered 2009 67 1 66 68 10.1159/000164400 18931511

[B27] König IR Validation in Genetic Association Studies Brief Bioinform 2011 12 3 253 258 10.1093/bib/bbq074 21546448

