# Supplementary material for: Genomic regions associated with bovine milk fatty acids in both summer and winter milk samples
Source: BMC Genet. 2012 Oct 29;13:93. doi: 10.1186/1471-2156-13-93 (PMC3536661; doi:10.1186/1471-2156-13-93)
Supplement: Additional 1 — Table S1. Most significant SNP per trait for each region significantly associated with fatty acids of the summer milk sample (corresponding to table 3), SNP position, significance level and the percentage of total additive genetic variance explained by the SNP. [file 1471-2156-13-93-S1.docx]

## Additional Table 1 – Most significant SNP per trait for each region significantly associated with fatty acids of the summer milk sample (corresponding to table 3), SNP position, significance level and the percentage of total additive genetic variance explained by the SNP

| Region | Trait | SNP | Chr | Position BTAU4 | Alternative Position | -Log_10_(*P*-value) | % of total additive genetic variance explained by SNP |
| --- | --- | --- | --- | --- | --- | --- | --- |
| 1a | C16:1 | ULGR_BTA-39422 | 1 | 106298619 |  | 3.84 | 3.31 |
| 1b | C14:1 | ULGR_BTA-58250 | 1 | 154751350 |  | 3.63 | 2.52 |
| 2a | C16:1 | ARS-BFGL-NGS-101408 | 2 | 65960161 |  | 5.03 | 4.03 |
| 2b | C18:1 | ULGR_rs29018764 | 2 | 113931092 |  | 4.54 | 5.02 |
| 2c | C16:1 | ULGR_BTA-93268 | 2 | 118914271 |  | 3.84 | 2.98 |
| 3 | C16:1 | ULGR_rs29027883 | 3 | 72584745 |  | 4.25 | 3.49 |
| 4a | C16:1 | ULGR_rs29015971 | 4 | 59974296 |  | 3.22 | 2.81 |
| 4b | C16:1 | ULGR_rs29024031 | 4 | 121815498 |  | 3.53 | 3.18 |
| 5a | C12:1 | ULGR_BTA-74162 | 5 | 9144954 |  | 4.69 | 4.02 |
| 5b | C16:1 | ULGR_rs29024155 | 5 | 35966378 |  | 3.88 | 3.37 |
| 5c | C6:0 | ULGR_AAFC03122217_7089 | 5 | 99946095 |  | 6.43 | 5.28 |
| 5c | C8:0 | ULGR_AAFC03122217_7089 | 5 | 99946095 |  | 5.36 | 4.91 |
| 5c | C10:0 | ULGR_BTA-61859 | 5 | 96448996 |  | 5.56 | 3.77 |
| 5c | C14:0 | ULGR_BTA-61859 | 5 | 96448996 |  | 4.16 | 2.78 |
| 5c | C10:1 | ULGR_rs29016908 | 5 | 101090417 |  | 4.24 | 3.22 |
| 5c | C14:1 | ULGR_BTA-74571 | 5 | 101084555 |  | 2.85 | 1.95 |
| 5c | C16:1 | ULGR_BTA-93285 | 5 | 96297427 |  | 3.15 | 2.65 |
| 5c | C18:1 | ULGR_AAFC03122217_7089 | 5 | 99946095 |  | 5.60 | 6.47 |
| 6a | C16:1 | ULGR_BTC-050897 | 6 | 40617870 |  | 3.57 | 2.89 |
| 6b | C6:0 | ULGR_BTC-038642 | 6 | 44772742 |  | 5.04 | 4.03 |
| 6c | C16:1 | BTA-76959-no-rs | 6 | 84955939 |  | 3.39 | 2.80 |
| 6d | C6:0 | ULGR_rs29012416 | 6 | 85288859 |  | 3.78 | 2.96 |
| 6e | C14:1 | ULGR_BTA-77644 | 6 | 106088917 |  | 3.66 | 2.86 |
| 7a | C16:1 | BTB-02031452 | 7 | 21946038 |  | 4.58 | 3.72 |
| 7b | C16:1 | ULGR_BTA-28678 | 7 | 64163271 |  | 3.69 | 2.96 |
| 10a | C10:0 | ULGR_BTA-105496 | 10 | 9818142 |  | 4.21 | 2.86 |
| 10b | C10:0 | ARS-BFGL-NGS-28483 | 10 | 22024690 |  | 5.16 | 2.99 |
| 10b | C12:0 | ARS-BFGL-NGS-28483 | 10 | 22024690 |  | 5.73 | 3.86 |
| 10c | C10:1 | ULGR_BTA-15583 | 10 | 89892045 |  | 3.85 | 3.13 |
| 11a | C16:1 | ULGR_AAFC03072692_59348 | 11 | 74307761 |  | 4.58 | 3.61 |
| 11b | C4:0 | ULGR_SNP_X14710_1740 | 11 | 107166278 |  | 6.00 | 5.46 |
| 11b | C6:0 | ULGR_SNP_X14710_1740 | 11 | 107166278 |  | 6.50 | 5.20 |
| 13 | C6:0 | ULGR_rs29027599 | 13 | 57042707 |  | 6.13 | 5.49 |
| 13 | C8:0 | ULGR_BTA-33016 | 13 | 57022323 |  | 4.96 | 4.64 |
| 13 | C10:0 | ULGR_BTA-33016 | 13 | 57022323 |  | 5.11 | 3.21 |
| 14a | C6:0 | ULGN_SNP_AJ318490_2 | 0 | 0 | sequence in DGAT1 | 17.66 | 14.05 |
| 14a | C8:0 | ULGN_SNP_AJ318490_2 | 0 | 0 | sequence in DGAT1 | 12.32 | 10.98 |
| 14a | C10:0 | ULGR_BTC-067569 | 14 | 3618555 |  | 6.84 | 4.24 |
| 14a | C14:0 | ULGR_SNP_AJ318490_1c | 14 | 445086 |  | 13.96 | 8.09 |
| 14a | C16:0 | ULGN_SNP_AJ318490_2 | 0 | 0 | sequence in DGAT1 | 47.81 | 47.41 |
| 14a | C10:1 | ULGR_BTC-068221 | 14 | 3011407 |  | 8.17 | 7.05 |
| 14a | C12:1 | ULGR_BTC-067762 | 14 | 3190704 |  | 5.21 | 4.55 |
| 14a | C16:1 | ULGR_SNP_AJ318490_1b | 14 | 445087 |  | 38.18 | 31.09 |
| 14a | C18:1 | ULGN_SNP_AJ318490_2 | 0 | 0 | sequence in DGAT1 | 45.82 | 50.13 |
| 14a | CLA | ULGR_SNP_AJ318490_1b | 14 | 445087 |  | 8.35 | 10.54 |
| 14b | C16:1 | ULGR_AAFC03000860_10938 | 14 | 45169820 |  | 3.90 | 3.57 |
| 14c | C16:1 | ULGR_AAFC03063557_87858 | 14 | 74282593 |  | 4.59 | 4.10 |
| 15a | C18:1 | ULGR_BTA-121008 | 15 | 20854340 |  | 3.91 | 3.61 |
| 15b | C16:1 | ULGR_BTA-37283 | 15 | 64123483 |  | 3.61 | 3.00 |
| 16a | C14:0 | ULGR_rs29019632 | 16 | 3732521 |  | 4.21 | 2.16 |
| 16b | C16:1 | ULGR_BTA-40002 | 16 | 68010217 |  | 3.63 | 3.03 |
| 17a | C6:0 | ULGR_BTA-19253 | 17 | 15030516 |  | 4.25 | 3.34 |
| 17a | C8:0 | ULGR_BTA-19275 | 17 | 15039540 |  | 4.36 | 3.90 |
| 17a | C16:1 | ULGR_BTA-40634 | 17 | 21040244 |  | 4.40 | 3.65 |
| 17b | C14:1 | ULGR_BTA-88832 | 17 | 24727021 |  | 3.76 | 3.00 |
| 17c | C6:0 | ULGR_BTA-40805 | 17 | 31496257 |  | 5.47 | 5.32 |
| 17c | C8:0 | ULGR_BTA-40805 | 17 | 31496257 |  | 7.61 | 8.18 |
| 17c | C10:0 | ULGR_BTA-40805 | 17 | 31496257 |  | 5.67 | 4.12 |
| 17d | C14:1 | ULGR_BTA-41023 | 17 | 50852615 |  | 3.84 | 3.26 |
| 17d | C16:1 | ULGR_BTA-41264 | 17 | 58681668 |  | 5.26 | 4.35 |
| 17e | C10:0 | ULGR_rs41255340 | 17 | 73990637 |  | 3.81 | 2.21 |
| 19a | C16:1 | ULGR_BTA-44680 | 19 | 6087775 |  | 3.43 | 2.86 |
| 19b | C8:0 | ULGR_rs41257373 | 19 | 58223746 |  | 6.40 | 6.03 |
| 19b | C10:0 | ARS-BFGL-NGS-24479 | 19 | 45901284 |  | 8.68 | 5.65 |
| 19b | C12:0 | ARS-BFGL-NGS-24479 | 19 | 45901284 |  | 8.23 | 6.29 |
| 19b | C14:0 | ULGR_BTA-45758 | 19 | 52099860 |  | 16.69 | 12.30 |
| 19b | C16:0 | ARS-BFGL-NGS-31468 | 19 | 46499482 |  | 4.19 | 4.28 |
| 20 | C6:0 | ULGR_BTA-50053 | 20 | 24052804 |  | 3.72 | 2.81 |
| 20 | C18:0 | BTB-00771394 | 20 | 9181457 |  | 6.13 | 15.59 |
| 20 | C16:1 | ULGR_AAFC03097520_1842 | 20 | 19545988 |  | 4.65 | 4.03 |
| 21 | C10:1 | ULGR_BTA-53024 | 21 | 65176950 |  | 5.19 | 4.24 |
| 22a | C14:1 | ULGR_BTA-55267 | 22 | 11843506 |  | 3.84 | 2.94 |
| 22b | C16:0 | ULGR_BTA-114990 | 22 | 16202758 |  | 4.10 | 3.68 |
| 23a | C16:1 | ULGR_BTA-55534 | 23 | 16383738 |  | 3.27 | 2.73 |
| 23b | C6:0 | ULGR_BTA-56106 | 23 | 27096170 |  | 5.16 | 4.34 |
| 23c | C16:1 | ULGR_AAFC03029112_9488 | 23 | 42701448 |  | 5.00 | 4.13 |
| 26 | C10:0 | ULGR_rs41255702 | 0 | 0 | 26:21139834 UMD3 | 7.62 | 4.53 |
| 26 | C10:1 | ULGR_rs41255702 | 0 | 0 | 26:21139834 UMD3 | 27.58 | 20.65 |
| 26 | C12:1 | ULGR_rs41255702 | 0 | 0 | 26:21139834 UMD3 | 18.63 | 14.98 |
| 26 | C14:1 | ULGR_rs41255702 | 0 | 0 | 26:21139834 UMD3 | 63.99 | 46.35 |
| 26 | C16:1 | ULGR_SNP_SCD | 0 | 0 | 26:21144708 UMD3 | 38.66 | 30.74 |
| 27 | C16:1 | BTB-00968596 | 27 | 47560218 |  | 3.28 | 2.63 |
| 28 | C8:0 | ULGR_BTA-107346 | 28 | 3124529 |  | 5.09 | 4.95 |
| 28 | C10:0 | ULGR_BTA-107346 | 28 | 3124529 |  | 4.72 | 3.09 |
| 29a | C10:0 | ULGR_BTA-22806 | 29 | 32659799 |  | 4.02 | 2.63 |
| 29b | C16:1 | ULGR_BTA-65824 | 29 | 44267475 |  | 5.42 | 3.55 |
| X | C14:1 | ULGR_BTA-30449 | 30 | 63061338 |  | 4.40 | 3.89 |
